# Host specificity in parasitic plants—perspectives from mistletoes

**DOI:** 10.1093/aobpla/plw069

**Published:** 2016-09-22

**Authors:** Desale Y. Okubamichael, Megan E. Griffiths, David Ward

**Affiliations:** 1 School of Life Sciences, University of KwaZulu-Natal, Private Bag X01, Scottsville, 3209, South Africa; 2 Plant Conservation Unit, Department of Biological Sciences, University of Cape Town, Private Bag X3, Rondebosch, 7701, South Africa; 3 Department of Biological Sciences, Kent State University, Kent, OH 44242, USA

**Keywords:** Bird dispersal, coevolution, geographic mosaic, haustorium, host compatibility, parasitic plants

## Abstract

Host specificity has been investigated for centuries in mistletoes, viruses, insects, parasitoids, lice and flukes, yet it is poorly understood. Reviewing the numerous studies on mistletoe host specificity may contribute to our understanding of these plants and put into context the dynamics at work in root parasitic plants and animal parasites. The mechanisms that determine host specificity in mistletoes are not as well documented and understood as those in other groups of parasites. To rectify this, we synthesized the available literature and analyzed data compiled from herbaria, published monographs and our own field studies in South Africa. As for other groups of parasites, multiple factors influence mistletoe host specificity. Initially, pollination affects gene flow. Subsequently, seed dispersal vectors (birds and marsupials), host abundance and compatibility (genetic, morphological, physiological and chemical), history and environmental conditions affect the interaction of mistletoes and their hosts and determine host specificity. Mistletoe–host network analyses and a geographic mosaic approach combined with long-term monitoring of reciprocal transplant experiments, genetic analyses of confined mistletoe populations and comparative phylogenetic studies could provide further insights to our understanding of host specificity. Some of these approaches have been used to study animal–plant interactions and could be adopted to test and evaluate host specificity in mistletoes at local and larger geographic scales.

## Abstract (precis)

Mistletoes are very fascinating parasitic plants. For centuries, people have been kissing under mistletoes during Christmas celebrations. Unlike most common plants, mistletoes grow on the branches of other plants and rely on these "host" plants for water and nutrients. Scientists have been trying to understand why parasitic plants differ in the number of host species parasitised. Like many parasitic plants, mistletoes can parasitise several plant species or only use one or far fewer species. This review contributes to the current broad understanding of parasite-host interactions using mistletoe as a case study. Future research needs are also highlighted.

## Introduction

Parasitic plants are very diverse (∼3500–4000 species) and display a considerable variation in host-specificity (Norton and Carpenter 1998; Norton and de Lange 1999; Thorgood and Hiscock 2010). However, our understanding of the evolution, ecology and speciation of host-specific parasitic plants remains limited (Ntoukakis and Gimenze-Ibanez 2016). Aerial parasitic plants—commonly called mistletoes, a term that describes a polyphyletic group of organisms with similar life histories—have also received little research attention because they generally cause less damage to commercial plants compared to root parasites (Yoder 1999; Mathiasen  *et al.* 2008; Ntoukakis and Gimenze-Ibanez 2016). Yet, they provide an opportunity to explore the origins of host specificity as a prerequisite for speciation in parasites. There are also substantial studies on mistletoe host specificity that can inform the broader study of plant and animal parasites. To link mistletoe studies with root parasitic plants and animal host specificity, a comprehensive review of our current understanding of host specificity in mistletoes is required.

Mistletoes are obligate hemiparasites that depend on hosts for water and nutrients and vary in their host use preference or specificity (Calder and Bernhardt 1983; Press and Graves 1995). Host specificity is the restricted use of available potential host species at a local scale, while host preference refers to the hierarchical ranking of host use (Thompson 1988; Norton and Carpenter 1998). For the purpose of this review, we view preference by mistletoes for particular hosts as a form of host specificity. In Africa, 70 % of mistletoes are generalist species that parasitize hosts from several families, 12 % are specific on hosts from one family but occasionally parasitize a few genera of other families and 18 % are specific to one or a few host species of a single genus (Polhill and Wiens 1998). The generalist mistletoes may encounter host species that vary in compatibility at different locality, thus may parasitize a subset of available host species at a given locality. This process could drive mistletoe specialization by selecting for host-specific adaptation at a local level (Norton and Carpenter 1998; Norton and de Lange 1999; Amico *et al.* 2007; Blick *et al.* 2012; Kavanagh and Burns 2012).

Mistletoes that are initially capable of utilizing several host species may also become restricted to a subset of available hosts within an area (Barlow and Wiens 1977; Amico *et al.* 2007; Okubamichael *et al.* 2011*a*; Kavanagh and Burns 2012, Lira-Noriega *et al.* 2015; Lira-Noriega and Peterson 2014). Generalist mistletoe species are therefore often composed of distinct host-specific populations (e.g. Lira-Noriega *et al.* 2015; Lira-Noriega and Peterson 2014). These host-specific populations of mistletoes could eventually speciate to produce host races (Norton and de Lange 1999; Jerome and Ford 2002; Lira-Noriega *et al.* 2015). Several factors such as seed dispersal vectors, host availability, host abundance, host compatibility and suitable niche for the parasite determine host specificity in mistletoes.

While a geographic mosaic approach is used widely to explain the relationship between specialization and coevolution, particularly within host–parasite associations in animals (*sensu* Thompson 1988, 1989, 1993, 1994, 1997, 2005*a*, *b*), it is rarely applied to mistletoe–host interactions. In this review, we propose a geographic mosaic approach that may help to explain mistletoe host specificity and at the end we suggest this to be integrated with the current understanding of host specificity.

## Mechanisms That Determine Host Specificity

Pollination by diverse mechanisms and seed dispersal mainly by birds initially affects the gene flow and the distribution of mistletoes on host trees. Thereafter, diverse host traits influence the establishment and survival of mistletoes, further filtering the distribution of mistletoes among host trees. Pollination of mistletoes is carried out by insects, birds and, rarely, by wind (Kirkup 1998; Tadey and Aizen 2001; Watson 2001). Thus, several different animal agents act as potential visitors to potential host trees. Self-compatibility in mistletoes is known to limit outcrossing and enhance inbreeding with nearby individuals (Vaknin *et al.* 1996; Ladley *et al.* 1997). In addition, differential flowering times among mistletoes may deter gene flow among species even in the same locality. For example, Amico *et al.* (2007) reported that peak flowering times of *Tristerix aphyllus* and the sympatric *T. corymbosus* do not overlap greatly, which limits interspecific pollen transfer. This is one of the factors believed to have influenced speciation in these two species in the genus *Tristerix*. *Arceuthobium americanum*, an obligate outcrossing species, also has limited pollen dispersal (maximum 400–512 m) and increases population differentiation (Jerome and Ford 2002). Preferential pollen transfer among individuals growing on the same or nearby trees may limit pollen flow among host races as well. Most importantly, natural hybridization of mistletoes is very rare and the success of a hybrid seedling would require an intermediate host that is unlikely to exist (e.g. *Amyema pendulum* and *A. quandang* produced only first-generation hybrids) (Bernhardt and Calder 1981; Calder *et al.* 1982). Natural hybridization is also almost absent in New World *Arceuthobium* (Hawksworth and Wiens 1996). Therefore, pollination acts as an important isolating mechanism for sympatric mistletoes.

Mistletoe species often rely on birds for direct seed dispersal (Aukema 2003). Only the dwarf mistletoes (*Arceuthobium* spp.) have seeds that are dispersed explosively (Hawksworth and Wiens 1996; Kelly *et al.* 2009), while *Misodendrum* is dispersed by wind (Vidal-Russell and Nickrent 2007) and *Tristerix* by marsupials (Amico and Aizen 2000). In bird-dispersed mistletoe species, the birds consume mistletoe fruits and subsequently wipe their bills, regurgitate or defaecate the seeds on the branches of host trees (Reid 1991; Aukema 2003; Roxburgh 2007; Green et al. 2009; Okubamichael *et al.* 2011*b*). Birds break the physical dormancy of the seed and initiate germination by removing the fruit cover (exocarp), which otherwise inhibits germination (Roxburgh 2007; Okubamichael *et al.* 2011*b*). The behaviours of the birds also expose the sticky viscin, enabling the mistletoe seeds to firmly attach to branches of host trees. Mistletoes and their dispersers have reciprocally evolved, although the nature of this coevolution is diffuse (see Reid 1991 and references therein). Some mistletoe species have developed contrasting colours of red, black, purple and dark blue that target specific frugivorous birds (Reid 1991). In turn, the avian genus *Dicaeum* (mistletoe birds) have modified tongues and crops that allow them to efficiently process mistletoe fruits (Reid 1991). They also frequently disperse the seeds to suitable host branches where the mistletoes can establish (Reid 1991). Similarly, members of the bird genus *Phainopepla* have a specialized digestive system and breed during the fruiting season of the desert mistletoes (*Phoradendron californicum*) (Walsberg 1975).

Bird dispersers determine the frequency of interaction of mistletoes with their hosts through the dispersal of their seeds to the host trees, and by determining the specific location of where seeds are placed on trees, which is key to mistletoe survival (Carlo and Aukema 2005). Birds usually disperse more mistletoe seeds on the parental host tree than elsewhere (Aukema and Martínez del Rio 2002*a*, *b*; Aukema 2003; Carlo and Aukema 2005). As with many other plant species, there is a negative correlation between mistletoe seed survival and dispersal distance from the parent plant (Okubamichael *et al.* 2011*b*). Birds are responsible for the local aggregation of mistletoes at a locality (infestation patches) or on individual trees (infection intensity) and determine a negative binomial distribution of mistletoes at the population level (Overton 1994; Robinson and Geils 2006). Marsupials like the Colocolo Opossum (*Dromiciops gliroides*) also had similar effects on aggregation in mistletoe (*T.corybosus*) infection that matched to their abundance in space in the temperate forests of Patagonia (García *et al.* 2009).

Dispersal by animal vectors determines mistletoe–host interactions across time and space, which in turn influences the geographic mosaic of mistletoes and their hosts. Birds are able to transfer mistletoe seeds across large distances and potentially disperse seeds to very distantly related hosts as well (Aukema and Martínez del Rio 2002*a*, *b*). Birds also potentially facilitate host switches by depositing mistletoe seeds on hosts that are not preferred. For example, in *T.* *aphyllus* and *T. corymbosus*, it is postulated that *T. aphyllus* arose from ancestral *T. corymbosus* seeds being deposited on a rare cactus host by mockingbirds that prefer high perches. Over time, some of these seeds became established and eventually developed reproductive isolation from *T. corymbosus* (Amico *et al.* 2007). Birds may also enhance the interactions between mistletoes and their prospective hosts, for instance, if the host trees provide a reward such as fleshy fruits that are available at the same time as the mistletoe fruits (Carlo and Aukema 2005; Okubamichael *et al.* 2011*b*). In either case, birds may potentially influence host specificity and host switches in mistletoes.

Birds may learn through time to differentially visit certain host species based on the reward of mistletoe fruits found only on infected trees (Godschalk 1983, 1985). The reward being offered increases the chance of efficient dispersal of mistletoe seeds to the appropriate host thereby facilitating host specificity (Martínez del Rio *et al.* 1995; Aukema and Martínez del Rio 2002*a*). Host specificity also enhances aggregation of individual mistletoes on trees of the specific host, which ensures that birds preferentially and constantly visit the same mistletoes and makes pollination frequent and easy (Watson 2011). In this regard, mistletoe species with high host specificity could be selected over those that are host generalists. Future research should investigate seed dispersal strategies of host generalist and specialist mistletoes by investigating fruit traits such as size, colour and nutritional quality. Specialist mistletoes would be expected to have fruit traits that target specific birds capable of directing seed dispersal to the appropriate host, thereby increasing fitness of the mistletoe species.

## Host Abundance and Compatibility

Diverse factors, but especially host traits, influence the establishment and survival of mistletoes and these traits further affect the distribution of mistletoes among host trees. In plant communities where species diversity is high and there are few dominant species, such as in rain forests, mistletoes tend to be generalists (Barlow and Wiens 1977). High host specificity is not likely to confer any selective advantage in such environments. Instead, there may be selection for traits that allow the mistletoes to infect and grow on a wide range of host species (Press and Graves 1995; Downey 1998). This clearly indicates that host abundance is an important trait that influences mistletoe host specificity. In less diverse temperate forests and semiarid savannas, where dominance of one or a few tree species is typical, mistletoes are more likely to be specific to one genus or even to a single host species (Norton and Carpenter 1998; Okubamichael *et al.*, 2011*a*). In these environments selection favours close physiological adaptations of the mistletoes to the predominant host species (Barlow and Wiens 1977; Dean *et al.* 1994; Downey *et al.* 1997; Downey 1998). There are many long-term associations of hosts and mistletoes that have evolved in a restricted, unidirectional way and that have resulted in extremely host-specific mistletoes (Norton and Carpenter 1998; Barlow and Wiens 1977). However, there are also instances where mistletoes parasitize uncommon host trees as a result of host compatibility at the genetic, mechanical, physiological and biochemical level (Yan 1993*a*; Yan and Reid 1995; Okubamichael *et al.* 2011*a*; Fadini 2011). This may create a geographic mosaic of mistletoe–host combinations across the landscape.

Usually the ever-changing composition of plant communities creates opportunities for new interactions between the mistletoe and host species (Thompson 1999). Thus, specialization may be a dynamic state capable of changing rather than being a static endpoint. Mistletoes have shorter generation times and higher reproductive rates than their host trees, which enable them to adapt quickly to a shift in host abundance in the ecosystem (Norton and Carpenter 1998). The shorter life cycle of the mistletoes may also facilitate a more rapid adaptation to host genotypes than the emergence of new resistance by host genotypes. When hosts develop resistance, selection would favour traits in the mistletoe that increase virulence or otherwise allow them to overcome host resistance or undergo host switching. This is largely consistent with the Red Queen Hypothesis or the evolution of an ‘arms race’ (van Valen 1973; 1976; Jokela *et al.* 2000; de Vienne et al. 2012). Specific research on this topic in mistletoes is lacking, and it would enhance our understanding of parasitism evolution in mistletoe.

The evolution of haustoria has enabled parasitic plants to acquire water and nutrients from other plants. It is suggested that parasitism in plants has evolved in arid environments where water and nutrients are limited (Atsatt 1973, 1977; Ehleringer *et al.* 1985; Bowie and Ward 2004). Nitrogen is often a limiting nutrient in plants, and mistletoes have been hypothesized to selectively parasitize host species that are high in nitrogen (Ehleringer *et al.* 1985; Midgley and Joubert 1991; Dean *et al.* 1994; Pennings and Callaway 2002). For example, Dean *et al.* (1994) found that mistletoe species richness was positively correlated with the average nitrogen level of the plant community in major vegetation types in South Africa. However, Griffiths *et al.* (2016) used a phylogenetically independent analysis of the Dean *et al.* (1994) data and found that the area occupied by a host species was more important than nitrogen in determining mistletoe species richness. This suggests that the quality of host trees in terms of nitrogen content may not be as critical as previously thought in terms of driving host specificity in mistletoes. This was even more pronounced in a global study by Scalon and Wright (2015) that showed nitrogen is not the limiting nutrient for mistletoes.

In any parasite–host association a parasite evolves traits that aid in effectively getting into the host. In response, the hosts usually build resistance to parasite infection (Thompson 1994, 2005*a*; Medel *et al.* 2010). Although in animals the relationship between immunology and resistance and susceptibility is known, comparable knowledge on parasitic plants and their host associations is absent. In mistletoes, the haustorium may encounter a range of resistance pressures by potential host trees, in which some individuals or host species are susceptible and some are resistant at various phases of haustorium penetration. The bark of many non-host plant species is resistant to haustorial penetration by mistletoes (Yan 1993*a*). Mistletoe infection could, through this process, be blocked before establishment can occur. For example, non-host species sometimes develop a wound periderm that blocks access to the xylem, thereby curtailing further establishment of mistletoes (Yan 1993*a*). Yan (1993*a*) showed that the primary host species of the mistletoes studied showed an initial bark resistance, which may be an important evolutionary adaptation to reduce infection. However, none of the primary host species exhibited xylem resistance. Thus, host trees may reject infection at different stages of mistletoe establishment by thwarting mistletoe penetration or blocking access to water and nutrients. There is also a suggestion (Hawksworth and Wiens 1996) that host trees could block the flow of xylem or phloem to infected branches, which obviously causes the death of the branch but protects the whole individual from nutrient drain to the parasite.

It is plausible to suggest that mistletoes could co-adapt with their hosts in the short-term and in the long-term and could co-speciate and shift hosts, but there are limited data to support this proposal. For example, Medel *et al.* (2010) recorded two cactus species (*Echinopsis chiloensis* and *Eulychnia acida*) having extremely long spines that deter infection by the mistletoe *T.* *aphyllus.* Mistletoe-dispersing Chilean mockingbirds (*Mimus thenca*) avoid perching on certain cactus hosts (*Echinopsis chiloensis* and *Eulychnia acida*) with extremely long spines (Martínez del Rio *et al.* 1995). Hence, host individuals with longer spines have lower mistletoe infection rates than those with shorter spines (Martínez del Rio *et al.* 1995). Even if birds disperse mistletoe seeds to long-spined cacti, the seeds remain hanging on the spine and their hypocotyl dies before it can form a holdfast on the host. In response, the mistletoes (*T.* *aphyllus*) have evolved a very long hypocotyl (the structure that protrudes as the mistletoe germinates and attaches to a host twig before forming a haustorium) that parasitize long-spined cactus hosts (Medel *et al.* 2010). However, such reports are rare in the literature on mistletoe–host coevolution. Therefore, host specificity can be used as a potential measure of coevolution (Thompson 1989).

At an early germination stage, mistletoe seeds are known for site- and host-insensitive nature, i.e. germinate quickly and indiscriminately on any substrate (Glazner *et al.* 1988; Yan 1993*b*; Rödl and Ward 2002). After germination, mistletoe survival depends on the successful attachment and penetration to the vascular tissue of the host tree. There is considerable evidence that mistletoe performance on different host trees varies. Clay *et al.* (1985) found that development of haustorial disks of *Phoradendron tomentosum* seedlings was significantly greater when experimental host and source host species were the same, rather than different, species. Okubamichael *et al.* (2014) also obtained a similar result demonstrating that hypocotyls of *Agelanthus natalitius* seedlings grew longer within their own source hosts. Dodder (*Cuscuta pentagona*), an aerial parasite, but not a mistletoe, also uses volatile chemicals released by the host to sense the location of the hosts and to cue haustorium development on preferred host species (Runyon *et al.* 2006). As yet there is not sufficient evidence on the role of volatile compounds and bark chemistry in directing host specificity in mistletoes. Even though the specific chemical interactions for mistletoes and their hosts are not known, many root parasites may require chemicals or a contact signal to recognize a host and initiate the development of the haustorium (haustorium- inducing factors (HIF), the flavonoids xenognosin A and B, quinone 2,6-dimethoxy-1,4-benzoquinone) (Jamison and Yoder 2001; Westwood *et al.* 2010) or they require a host chemical signal for germination (Strigolactones) (Xie *et al.* 2010; Ćavar et al. 2015). Tomilov *et al.* (2006) indicated that HIFs might be species specific and activate specific receptors in particular parasites or host plants that may produce several HIFs with possible redundancy of active molecules.

## Geographic Mosaic Approach

Coevolution is a reciprocal evolutionary change in interacting species at local, regional and global levels that is driven by natural selection, creating ever-changing geographic mosaics of species interactions with one another (Thompson 1989, 1994, 2005*b*). Thompson (2005*b*) argues that the coevolution between pairs of species or populations within a local scale must be maintained to eventually establish the interaction across a broader geographic range. Thompson (1997, 2005*b*) describes this as a geographic mosaic model and proposes that coevolving interactions, which collectively drive ongoing coevolutionary dynamics of global biodiversity, incorporate three components: *geographic selection mosaics*, *coevolutionary hotspots* and *trait remixing.*

Genotype-by-environment interactions determine the fitness of interacting species across regions. Natural selection acts on this variation causing population specialization in different regions, which is referred to as *geographic selection mosaics*. *Coevolutionary hotspots* are subsets of communities in which much of the evolutionary change occurs where local selection is non-reciprocal. Such coevolutionary hotspots are also often embedded in a broader matrix of coevolutionary coldspots (Gomulkiewicz *et al.* 2000). The geographic range of a parasitic species may only overlap with that of its preferred host(s) at certain localities. The three-way interaction between mistletoe–bird–host (Reid 1991; Aukema and Martínez del Rio 2002*a*, *b*) may create coevolutionary hotspots in which certain local populations contribute greatly to the overall coevolution between the mistletoes and their hosts. This could create a mosaic of mistletoe–host association, which varies through space and time. *Trait remixing* occurs through changes in the genetic structure of coevolving species due to mutations, gene flow, random genetic drift and extinction of local populations. The continuous altering of the spatial distributions of potentially coevolving genes and traits often drives the processes of coevolution (Thompson 2005*b*).

The works of Lira-Noriega *et al.* (2015) and Lira-Noriega and Peterson (2014) on the mistletoe *Phoradendron californicum* have demonstrated that the distribution of this species is strongly affected by the evolutionary hot spots where the host, dispersal vectors and environmental condition of the mistletoes overlap. Their studies highlight the importance of the geological past interacting with the mistletoe–host associations in structuring phylogeography and initiating host races in mistletoes. Using herbarium voucher specimens, Lira-Noriega and Peterson (2014) tested three niche hypotheses (host, vector and parasite) that likely mediate mistletoe distribution and found that host availability alone does not determine mistletoe establishment. Instead, suitable environmental conditions for the mistletoe are a prerequisite. Jerome and Ford (2002) showed that *Arceuthobium americanum* has at least three distinct genetic host races that potentially could undergo speciation with time. Most importantly, these studies clearly demonstrate that a geographic mosaic approach can explain the mosaic nature of the distribution of mistletoes and patterns in host specificity.

## Host Specificity in South Africa from Literature, Herbaria and Field Observations

**Figure 1:**
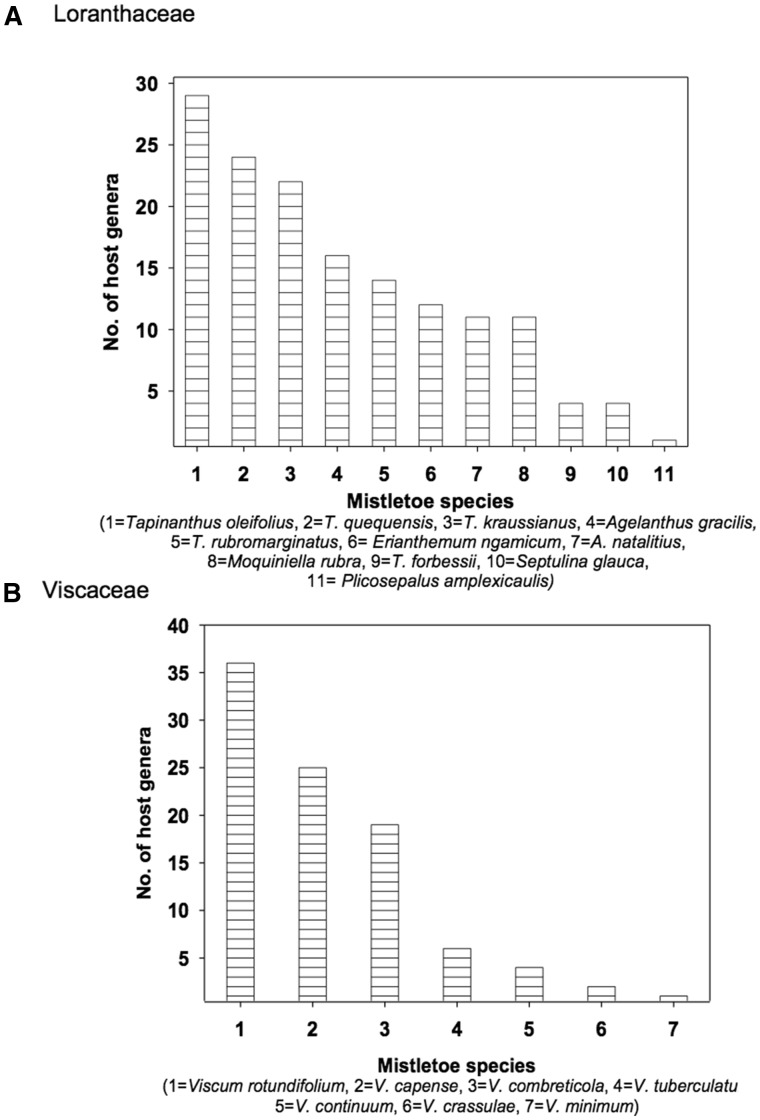
The number of parasitized host genera by the prospective mistletoe species (data modified from Visser, 1981). This summarizes the pattern of infection of the common mistletoe species found in southern Africa of the two largest families of mistletoes: (A) Loranthaceae and (B) Viscaceae.

It is important to investigate host specificity at the family, genus and species levels because the available species that can be parasitized at particular levels vary, and not all species in a genus can be parasitized equivalently by specialist mistletoes. Unfortunately, monographs for mistletoes in Africa only report host use mainly at the genus level (Polhill and Wiens 1998; Wiens and Tolken 1979; Visser 1981). Analyses of Shannon-Wiener indices (H') of data from the mistletoe literature (Polhill and Wiens 1998; Wiens and Tolken 1979; Visser 1981) show that the two main mistletoe families in southern Africa parasitize a high diversity of host genera. Mistletoes from Loranthaceae parasitize 89 host genera with H'  = 4.26, while those in Viscaceae parasitize 65 host genera with H'  = 4.05 (Fig. 1). Many of the southern African mistletoes in these two families use *Acacia* and *Combretum* as their main host plants. A few mistletoe species are very host-specific and use only one genus over their entire geographic range (e.g. *Viscum minimum* parasitizes only *Euphorbia horrida* and *E. polygona* in the Eastern Cape, South Africa) (Polhill and Wiens 1998).

**Figure 2:**
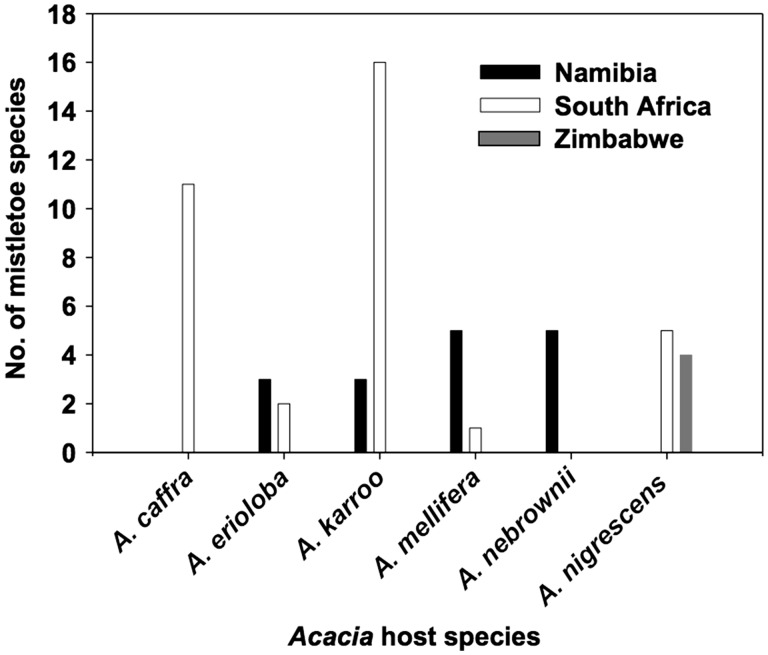
Number of mistletoe species that parasitize the most common *Acacia* host species in southern Africa. *Acacia karroo* is the most abundant host tree in South Africa and many types of mistletoe species utilize this abundant species. However, in Namibia, *A. erioloba* and *A. mellifera* are quite common and were the most common hosts for mistletoes. In Zimbabwe, *A. nigrescens* is common and is also highly utilized by mistletoe species in the area (see van Wyk and van Wyk 1997 for the distribution pattern of each *Acacia* species).

Using specimens from the Bews Herbarium at the University of KwaZulu-Natal, we compiled data to give us a more comprehensive understanding of host use by mistletoes that can be traced to the species level [**see Supporting Information—Appendix 1**]. The collection includes 340 herbarium specimens of mistletoes from Loranthaceae (46 mistletoe species recorded from over 200 host species), and 179 herbarium specimens of mistletoes from Viscaceae (14 mistletoe species recorded from over 70 host species). From the herbarium investigation it is clear that *Acacia karroo* and *A. caffra* were the most commonly used host species in South Africa (Fig. 2). This may be related to the availability of these *Acacia* species, as *A. karroo* is the most widely distributed *Acacia* species in South Africa (van Wyk and van Wyk 1997). However, in areas where *A. karroo* is not the most abundant potential host species, many mistletoe species are found on other host species (Fig. 2). *Viscum rotundifolium* was found parasitizing the highest number of host species in KwaZulu-Natal, but it was restricted to *Ziziphus mucronata* in the Free State and Northern Cape provinces in South Africa (see also Okubamichael *et al.* 2011*a*). The same species was found on the more abundant host species *Boscia albitrunca* and *B. foetida* in Namibia. These results support the hypothesis that mistletoe species that are host generalists across the entire range can be specific to particularly abundant hosts on a local scale (e.g. *Agelanthus natalitius*, *Phoradendron leucarpum*, *Arceuthobium globosum* and *Viscum album*). Norton and de Lange (1999) found that this pattern is also common in New Zealand mistletoes (*Alepis flavida*, *Peraxilla tetrapetala* and *P. colensoi*) that parasitize only the abundant species of *Nothofagus*.

Herbarium records showed that many mistletoe species tend to have one primary host species and use other host species less frequently, at least in the South African collections investigated. Even in the most generalist mistletoes, not all available host genera are equally susceptible to infection by mistletoes at a given locality. Usually mistletoes have a primary host genus that they prefer or on which they become host-specific. This may be linked to the existence of coevolutionary hotspots where the interactions between mistletoes and their hosts are strong. The mistletoes *Plicosepalus kalachariensis* and *P. undulatus*, for instance, parasitize only *Acacia* species and may provide good examples of mistletoe coevolution. *Viscum menyharthii* also parasitizes predominantly *Acacia* and *Ficus* species, even though *Acacia* species are generally not the primary hosts for Viscaceae.

We also found that mistletoe species are less likely to share a single primary host genus, especially if they are from different families. A Sørensen index (*Sim*) was used to calculate the similarity in host genera use by the two major mistletoe families. This index is calculated as 2C/A + B, where A = number of species in sample A, B = number of species in sample B and C = number of species common in both A and B (see e.g. Magurran 1988). The Sørensen index comparing the host species used by mistletoes in the Viscaceae and Loranthaceae was low (*Sim* = 0.26) with only 20 host genera shared between Loranthaceae and Viscaceae. This indicates that mistletoe species in Viscaceae parasitize mainly host genera that are not used by mistletoe species in Loranthaceae and *vice versa*. For example, *Euphorbia* and *Olea* are some of the most common host trees for mistletoes in Viscaceae but they are not common hosts for mistletoes in Loranthaceae. Additionally, even the most generalist mistletoe species in Loranthaceae (*Tapinanthus oleifolius*) and the most generalist mistletoe in Viscaceae (*Viscum rotundifolium*) had a low similarity index for host use (*Sim* = 0.29). *Viscum rotundifolium* does not utilize all 32 species of *Acacia* that are reported to be parasitized by other mistletoes in southern Africa, but instead only occurs on *A. erioloba* and *A. karroo*. These findings show clear trends for southern Africa that could be further tested by examining host ranges in these two mistletoe families in North America and Australia.

**Figure 3:**
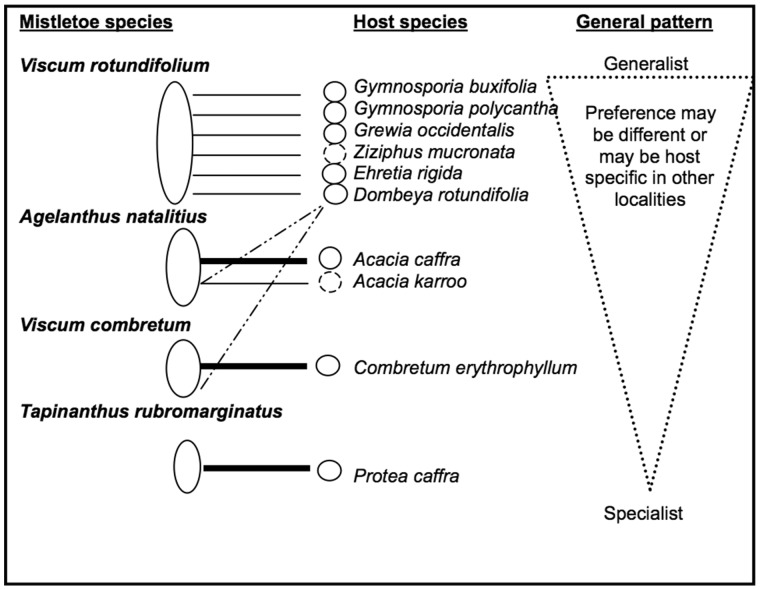
We recorded four mistletoe species that reflect the general pattern of host specificity of mistletoes at Walter Sisulu National Botanical Garden, Johannesburg, South Africa. The mistletoes differ from being generalist to host-specific at the site. *Viscum rotundifolium* was the generalist mistletoe species that parasitizes at least six tree species, but it does not appear to parasitize tree species that are sole hosts for other co-occurring mistletoes. *Agelanthus natalitius*, has a limited number of host species and predominantly parasitizes *Acacia caffra.* It is more rarely found on *Dombeya rotundifolia* and *Acacia karroo*. *Viscum combretum* mainly parasitizes *Combretum erythrophyllum* and rarely is found on *Dombeya rotundifolia*. At the extreme end of host specificity, *Tapinanthus rubromarginatus* parasitizes only *Protea caffra.* Dashed circles of host trees indicate that they are rare at the location. Dashed lines that link the mistletoe–host interactions indicate that the associated mistletoe seldom parasitizes those host trees. The broader and darker lines indicate mistletoes that are specific to the indicated host trees. The triangle shows that the mistletoes range from host generalist (indicated by the base of the triangle) to host-specific (indicated by the pointed end of the triangle) species.

We have also observed that several mistletoe species in the Walter Sisulu Botanical Garden (near Johannesburg, South Africa, ca. 300 hectares) have a non-overlapping domain of host species. In this particular site, if a host species is parasitized by a particular mistletoe, it is unlikely to be parasitized by other mistletoe species occurring in the same habitat (Fig. 3). A negative co-occurrence pattern in mistletoe species that specialize on distinct suites of host species has been also reported in North America, New Zealand and Australia (Hawksworth and Wiens 1972; Blick and Burns 2009; Blick *et al.* 2012). Similarly, Fadini (2011) showed that three congeneric and sympatric mistletoe species (*Psittacanthus biternatus*, *P. eucalyptifolius* and *P. plagiophyllus*) specialize on different host species in the Amazon. Mistletoes that are host-specific may have a competitive edge over non-specific mistletoes where several mistletoes coexist. For example, Jerome and Ford (2002) suggested that host specificity in *Arceuthobium americanum* reduces potential competition with other mistletoe species that may utilize the same host species at a given site. A pattern of non-overlap in mistletoe primary host use may be indicative of competitive exclusion and could contribute to a geographic mosaic of mistletoe–host interactions. Such a geographic mosaic could ultimately determine patterns of host specificity in mistletoes (*sensu* Blick and Burns 2009). Further investigation is warranted to quantify the degree of competition among mistletoe species and to determine the mechanisms that drive such interactions.

At present, it is clear that there is a need to understand the complex networks of mistletoe–host interactions (Vidal-Russell and Nickrent 2008). Ecological networks most frequently fit nested or modular patterns (Genini *et al.* 2012). Networks that are nested contain a few generalists that interact with one another and with specialist species, which allows for the persistence of specialists. In modular networks, generalist species form sub-groups (modules) that interact more with the species within their module than they do with species in other modules. Network analysis (a test of modularity and nestedness) could be used to examine the structure of patterns of mistletoe–host interactions at the population, species, genera and family levels (*sensu* Genini *et al.* 2012) [**see Supporting Information—Appendix 1**]. This should be supplemented by a more comprehensive reciprocal transplant analysis and a genetic study. Together, these investigations could reveal the underlying processes that are responsible for the development and maintenance of mistletoe host specificity.

## Future Directions

It would be useful to test whether the geographic specialization of mistletoes on different hosts results from genetic divergence in preference hierarchies (phylogenetic host specificity) or ecological differences in the availability of hosts (specificity in geographic space). For example, it is well known in animal parasites that some populations exclusively parasitize one host for many generations but do not lose their ability to recognize other major hosts that they do not normally encounter (Poulin 2010; Poulin *et al.* 2011; Cooper *et al.* 2012). On the other hand, some animal parasites switch to new hosts and lose their ability to infect the host species that previously acted as a host. Currently, there are no data on this subject in mistletoes and it would be important to use a similar line of investigation to examine coevolution in mistletoes and their host trees. On an evolutionary time scale, mistletoes switch among different host species and the haustorium or other traits (e.g. stomata) probably requires adaptive plasticity so that they can access nutrients and water from the prospective host species (see Gonzáles *et al.* 2007).

It would be ideal to test the geographic mosaic model using reciprocal transplant experiments on a range of host species and sites to determine differences in mistletoe fitness on different hosts (such as haustorium establishment, survival and reproduction). Reaction norms, which are the pattern of phenotypes produced by a given genotype under different environmental conditions, could then be used to determine the selection pressure in populations of mistletoes in different environments (Yan 1993*b*; Lynch and Walsh 1998; Rödl and Ward 2002). Reciprocal transplant experiments on mistletoes tend to result in low establishment success (Rödl and Ward 2002), which require using large sample sizes. Molecular markers could also be used to investigate genetic differentiation among populations. For example, host race speciation in *Tristerix* (*T. corymbosus* to cacti-specific *T. aphyllus*) was supported using molecular phylogenetic methods (Amico *et al.* 2007; Amico and Nickrent 2009).

A phylogenetic comparison of mistletoes and their hosts could reveal the relative importance of coevolution and host-switching events in mistletoe speciation (see Jerome and Ford 2002). It would be important to determine whether mistletoes parasitize phylogenetically or biogeographically similar hosts. The combined results of these investigations would comprehensively test the geographic mosaic model in order to explain the mistletoe–host interaction at local and at larger geographic scales.

## Source of Funding

The National Research Foundation of South Africa and the Claude Leon Foundation provided funding for this research.

## Contributions by the Authors

D.Y.O. reviewed the available information, and collected and analyzed the required data for the paper. D.W. and M.E.G. provided the supervision and contributed in conceptualization of the synthesis.

## Conflicts of Interest Statement

None declared.

## Supplementary Material

Supplementary Data
