# Supplementary material for: Host specificity in parasitic plants—perspectives from mistletoes
Source: AoB Plants. 2016 Sep 22;8:plw069. doi: 10.1093/aobpla/plw069 (PMC5206351; doi:10.1093/aobpla/plw069)
Supplement: Supplementary Data [file supp_8_plw069_index.html]

Sign In 

**This item requires a subscription\* to AoB PLANTS.**

If you would like to access this item you must have a personal account. Please sign in below with your personal username and password or  Register  to obtain a username and password for free.

Oxford Journals Subscribers and Registrants Sign In

If your subscription is through Oxford University Press, or you have signed up for personalization on this site, sign in below.

Sign In Username  Password    Remember my username & password.

Forgotten your username or password?

- Can't get past this page?
- Help with Cookies.
- Need to Activate?

OpenAthens Users

- Sign in via OpenAthens : If your organization uses OpenAthens, you can log in using your OpenAthens username and password. Contact your library for more details.
- List of OpenAthens registered sites, including contact details.

Login via Your Institution

- Login via your institution : You may be able to gain access using your login credentials for your institution. Contact your library if you do not have a username and password.

Register or Subscribe

- Subscribe to the Journal - Subscribe to the print and/or online journal.
- Register  - Register online for access to selected content and to use Pay per View. Registration is free.
